# Characterization of mechanical properties for tubular materials based on hydraulic bulge test under axial feeding force

**DOI:** 10.1016/j.fmre.2022.01.024

**Published:** 2022-02-12

**Authors:** Bin Zhang, Benny Endelt, Karl Brian Nielsen

**Affiliations:** a Department of Materials and Production, Aalborg University, Aalborg 9220, Denmark; b Engineering Teaching Practice Training Center, Tiangong University, Tianjin 300387, China; c Research and Development Lab, Vestas aircoil A/S, Lem 6940, Denmark; d Department of Mechanical and Production Engineering, Aarhus University, Aarhus 8000, Denmark

**Keywords:** Intelligent optimization, Tubular material, Parameter identification, Hydraulic bulge test, Constitutive model

## Abstract

A T-shape tube hydraulic bulge test under axial feeding force is carried out to characterize the mechanical properties of EN AW 5049-O and 6060-O aluminium alloys. The punch displacement, T-branch height and axial compressive force are recorded online during the experiment. An intelligent inverse identification framework combining the finite element method and numerical optimization algorithm is developed to determine material parameters by fitting simulated results to the experimental data iteratively. The identified constitutive parameters using the inverse modelling technique are compared with those determined by the theoretical analysis and uniaxial tensile test. The comparison shows that the predicted bulge height and punch force based on the material parameters obtained by the three methods are different and the inverse strategy produces the smallest gap between numerical and experimental values. It is possible to conclude that the hydraulic bulge test can be applied to characterize the stress-strain curve of tubular materials at the large strain scope, and the automatic inverse framework is a more accurate post-processing procedure to identify material constitutive parameters compared with the classical analytical model.

## Graphical abstract

**Figure:**
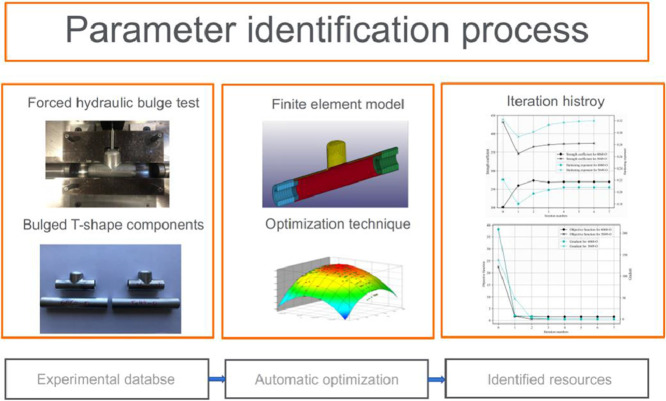

## 1 Introduction

Tube hydroforming technologies are playing an increasingly important role in modern advanced manufacturing processes, which provide more possibilities for lightweight design and precision production of complex tubular components used in the automotive and aerospace industries [1], [2]. The stable quality and excellent performance of tubular products in the metal forming processes require essential information such as the hardening and fracture of the incoming metallic tube [3], [4]. Further, an accurate output from the finite element (FE) method also depends heavily on the reliable mechanical property characterization [5], [6], [7].

Scientists and engineers have proposed many different experimental methods to characterize the mechanical properties of thin-walled tubes. Tensile tests are carried out for the specimens cut from tubes along the longitudinal direction, but this operation is difficult to achieve on tubes with small diameters [8], [9]. Ring samples can be cut from small thin-walled tubes along the circumferential direction [10], [11]. However, the stress and strain state of these samples in the longitudinal or circumferential tension test is quite different from that in the actual tube forming process where the equivalent plastic strain is in the range of 1-1.5 and dominated by compression whereas the uniaxial data is in the range of 0.15 to 0.4 depending on the material [9]. Thus, FE models depend heavily on extrapolation of the hardening behavior, and such an extrapolation can reduce the prediction accuracy from FE outputs [12].

Axial and lateral compression tests of the whole tube can be performed to determine the flow stress curve of the tubular material under compressive stress states and without machining specified shaped samples, which are described in these works [13], [14], [15]. One drawback of these testing methods is that the sudden buckling of tubular specimens causes unstable and incomplete data collection during the experiment and this test is only limited to determining material behaviour in one direction, which may lead to large errors for FE simulations in some cases [16]. Compared with the above experimental methods, the tube hydraulic bulge test is a more advanced characterization technique that can comprehensively represent the mechanical properties of tubular materials with flexible end-conditions [17].

Many researchers have investigated different types of hydraulic bulge tests but most of them focus on tube hydro bulging processes with free or fixed end-conditions [18], [19], [20], [21], [22], [23], [24]. Various experimental setups have been designed and manufactured to carry out the above experiments. For instance, Fuchizawa et al. [19] have designed a bulging device where tube ends are locked on the end supports to seal the internal liquid, and one of the supports can move freely to reduce the longitudinal stretching of specimens. Koc et al. [20], [25], [26] use a stand alone press to bulge tubular materials, in which both ends of the tube are completely fixed by the friction between dies and urethane expansion plugs. Zhang et al. [27] apply a more flexible locking system to restrain the axial movements of samples, and tubes with different diameters and wall thicknesses can be tested by replacing gaskets. Another design mechanism has been proposed [22] to achieve a fixed end-condition, where conical punches with an angel are used to form tube ends with the wedge expansion shape to avoid the axial sliding of tubular samples. However, free or fixed bulge tests without axial feeding force will reduce the bulge height and the equivalent strain in a small range can be obtained.

On the other hand, a number of efforts have been made in the modelling of tube hydraulic bulging processes with fixed and free end-conditions to determine the flow stress curve of tested tubular materials. These developed theoretical models are based on membrane theory where a force equilibrium equation is constructed on the thin element at the center of the bulge deformation zone using the plane stress hypothesis [28]. Strain components are calculated based on the volume constancy law and different geometrical assumptions for the bulge profile shape such as a circular arc [19], two circumference arcs [23], [29], an eplliptical curve [30], [31] and a spline function [21]. A detailed comparison of advantages and drawbacks for the above models is presented in these works [32], [33], [34], and isolated stress-strain solutions and excessive assumptions can reduce the accuracy of identified results using the above methods. It should be pointed out that the inverse strategy has made substantial progress in accuracy improvement of determined parameters for tubular materials [27], [35]. However, the application cases of the inverse scheme focus on the free and fixed hydro bulging processes [24], [27], [36], [37], [38], [39], and fewer papers have reported on the inverse strategy applied to the tube hydraulic bulge test under axial compressive force.

In this study, T-shape hydraulic bulge tests under axial feeding force for two types of thin-walled metal tubes have been performed on a multifunctional hydraulic machine. An inverse framework combining the FE method with the Levenberg-Marquardt algorithm is used to identify material parameters based on the experimental data collected from bulge tests. The paper structure is as follows: Section 2 presents all the experimental work including tested tubular materials and experimental tools. The inverse strategy and theoretical analysis to determine the parameters of tubular materials are introduced in Section 3. In Section 4, the experimental data and results comparison of identified material parameters obtained by different methods are discussed. The main conclusions are drawn in Section 5.

## 2 Experimental work

### 2.1 Tested material

Hydraulic bulge tests were carried out for the thin-walled seamless tubes made of EN AW 5409-O and EN AW 6060-O aluminium alloys. 5049-O aluminium is widely applied to the air cooling and heat exchanger system of an automotive because of its excellent formability and corrosion resistance [40]. The latter material is a common commercial aluminium alloy used in civil and architecture engineering [41]. In the current study, the used tubular samples were fully annealed before the actual experiments and their initial nominal wall thickness and external diameter are 1.50mm and 32.00mm, respectively. Tested specimens are cut into 150.00mm length from the same tube batch to reduce unpredictable errors.

### 2.2 Experimental setup

**Fig. 1:**
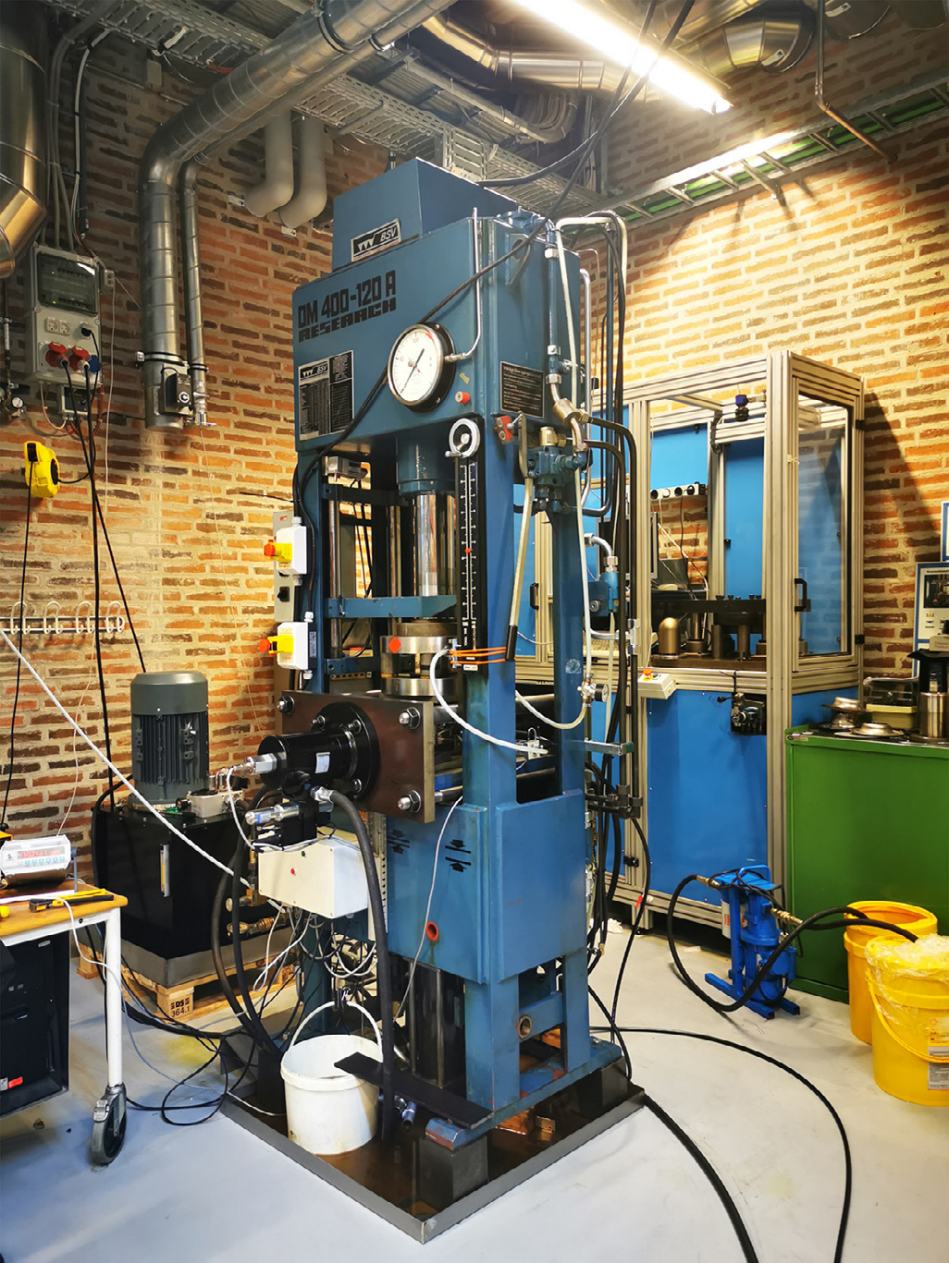
**Experimental setup for tube hydraulic bulge test**.

**Fig. 2:**
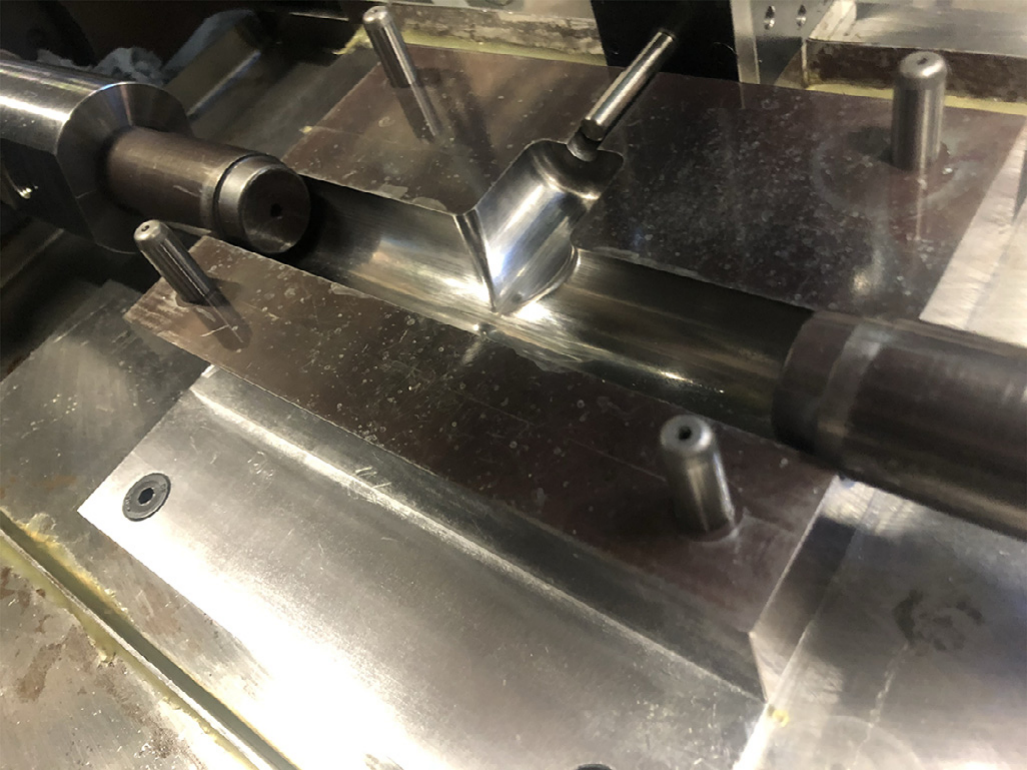
**Dies and axial punches in experimental tools**.

A special hydraulic press is developed to perform the tube hydraulic bulge test under axial feeding force for the above two materials. Fig. 1 presents an overall view of this designed experimental equipment. The hydroforming machine consists of the pressure system, clamping devices, tools and the control system, in which a short-stroke vertical hydraulic cylinder is used to provide enough closing force to lock the T-shape die during the forming process. The longitudinal feeding force is provided by two axial hydraulic cylinders that control the positions of two punches at the same time. Two punches can be easily replaced with various sizes and dimensions to achieve a more flexible test. The shape of the axial punch ends is a tapered curve to avoid fluid leakages and pressure losses, which is shown in Fig. 2. The combined utilization of a low-pressure pump and intensifier can deliver sufficient internal pressure, up to 80MPa in the current research.

**Fig. 3:**
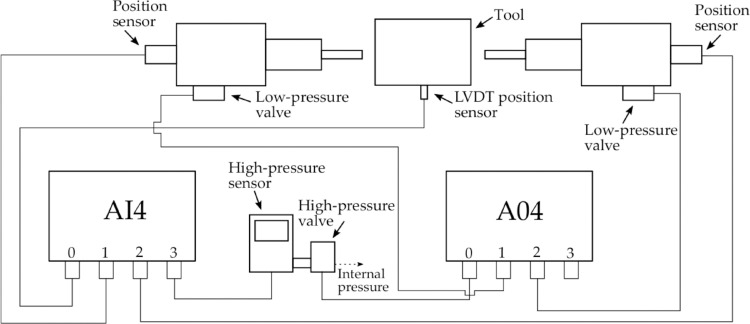
**Flow chart of the overall electrical system for hydraulic press**.

To measure the axial displacement precisely, a digital position transducer is installed on the punch end, which can record the accurate coordinate of the axial punch during the forming process. At the same position, a force transducer is integrated into it to acquire the punch force at different stages. The sensor connected to a high pressure valve can measure the internal pressure in the tube and the application of the closed-loop control system enables the actual fluid pressure to reach the predefined value. A linear variable differential transformer position sensor is used to collect the filling height of the tube branches online. Fig. 3 shows a flow chart of the electrical system for this hydraulic press. All operations including data acquisition and process control will be completed on an industrial computer running the GNU/Linux operating system.

### 2.3 Tube hydraulic bulge test

**Fig. 4:**
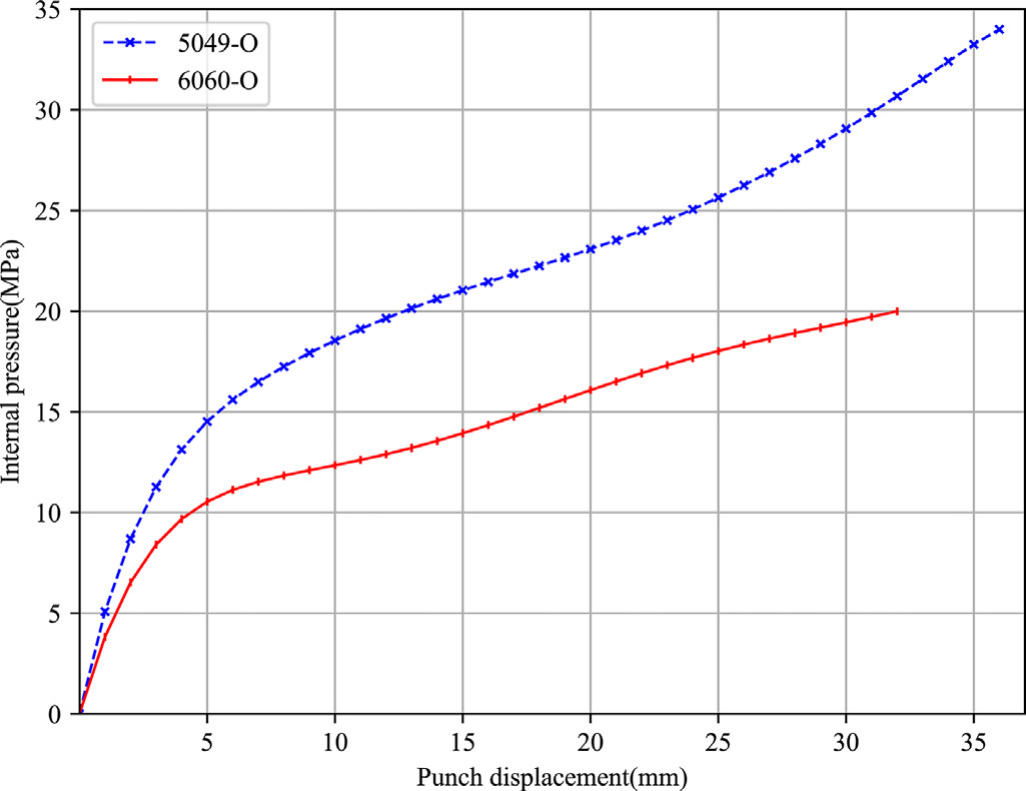
**Approximate loading path for 5049-O and 6060-O aluminium alloy in hydraulic bulge test**.

The T-shape tube hydraulic bulge test for aluminium alloys 5049-O and 6060-O is conducted on the machine shown in Fig. 1. A complete experimental process can be divided into two stages. In the first step, the objective is to generate a reasonable loading path, i.e., axial displacement versus internal pressure for the hydraulic bulge test. Several potential loading paths obtained by an automatic optimization program are tested on the actual press until a perfect tubular component that has no wrinkles and fractures is produced. Furthermore, three tubular samples are repeatedly tested using the selected loading path to ensure its effectiveness and robustness. Fig. 4 presents the optimized loading path for two aluminium alloys.

In the second stage, thin-walled aluminium alloy tubes are bulged under internal fluid pressure and axial compressive force. The loading path follows the one obtained in the first stage, and the feedback control system can guarantee that the actual loading path is consistent with the definition. The experimental data, i.e., filling height, axial feeding force and punch axial displacement, is recorded online by the data acquisition system while the pole thickness at the tube center is manually measured after the deformation.

### 2.4 Tensile test

**Fig. 5:**
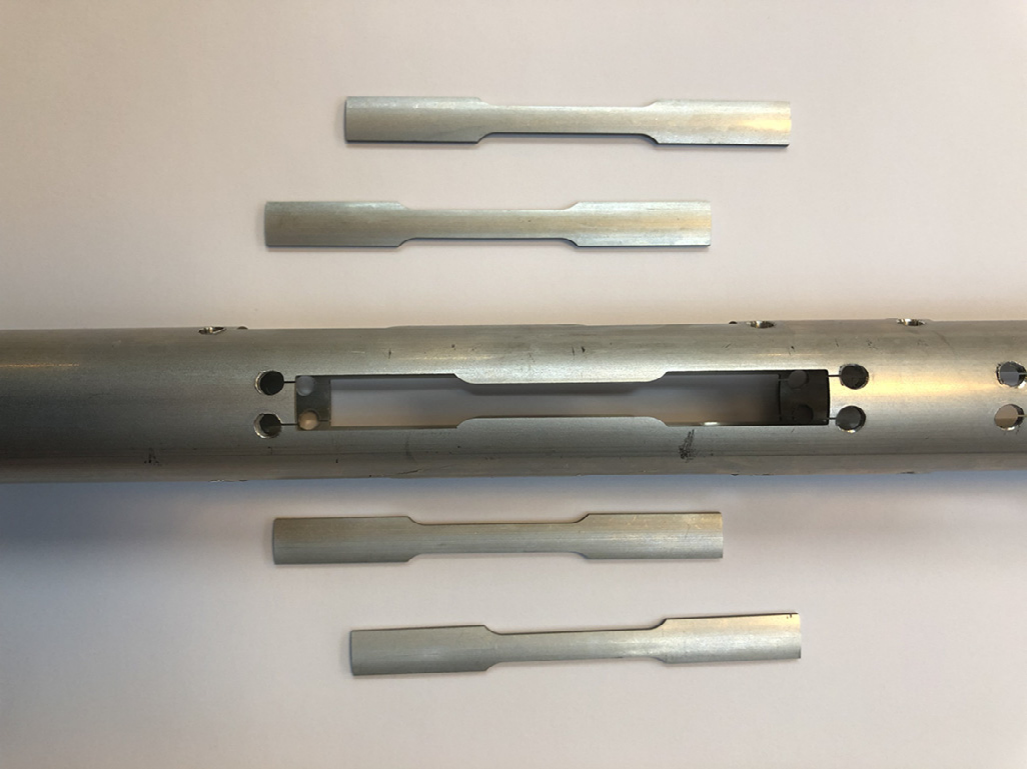
**An illustration of how tensile specimens are cut from the tested tube at different circumferential positions**.

The universal tensile test is conducted to determine the mechanical property and flow stress curve of used tubular materials. Tension specimens are cut at four different positions, i.e., $0^{\circ },90^{\circ },180^{\circ },270^{\circ }$ along the circumferential direction on the used tubes. Their sizes and dimensions follow the ASTM E8 standard [42]. Fig. 5 illustrates how tension specimens are cut from the tubular materials in the longitudinal direction. All tests are performed on an electrical universal testing machine from Instron corporation and the punch velocity is 1.4mm/min at room temperature. A mechanical extensometer is used to measure the deformation data like the displacement and load of tension samples during the test. All collected experimental data will be used to determine the parameters of tubular materials.

## 3 Parameter identification method

**Fig. 6:**
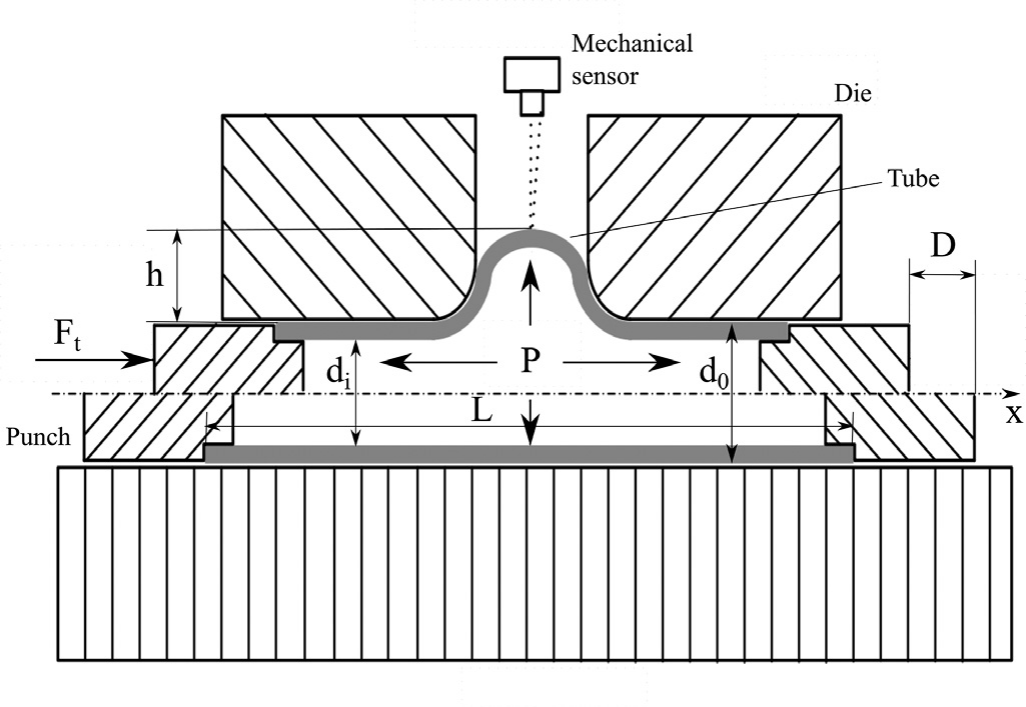
**Schematic diagram of T-shape tube hydraulic bulge test**.

The T-shape hydraulic bulge test is an experimental characterization method to describe mechanical properties of tubular materials, which can reproduce the actual process condition in a tube hydroforming operation and generate the basic database for the identification of material constitutive parameters. Fig. 6 illustrates the schematic diagram for the T-shape tube hydraulic bulge test. Various analytical and numerical models are developed for the bulging process and their advantages and limitations are presented [32], [34]. In the following sections, two types of models are used as post-processing procedures to identify material constitutive parameters. One is an analytical model based on energy theory, and the other is an inverse model combining FE simulations with gradient-based algorithms.

### 3.1 Analytical model

The classical slab method is widely used for the modelling of hydraulic bulge tests of axisymmetrical tubular components, which can determine the stress-strain curve analytically by solving a force equilibrium equation defined on a small element of hydroformed parts [26]. However, this theoretical approach can not analyze and describe the T-shape tube hydraulic bulge process because the final hydroformed parts are not axisymmetrical and angled branches increase the complexity of boundary conditions. The energy theory provides a possibility to analyze the T-shape tube hydraulic bulging process without considering force balance equations and boundary conditions.

As shown in Fig. 6, the T-shape tube hydraulic bulging process can be considered as a plane strain case where the strain component in the longitudinal direction can be neglected, i.e., $\varepsilon_{x}=0$. From the condition of volume constancy, the radial and circumferential strain on the branch center can be calculated as

(1) $$\begin{array}{cc} & \varepsilon_{t}=\ln\frac{t}{t_{0}} \end{array}$$

(2) $$\begin{array}{cc} & \varepsilon_{\theta }=-\left(\varepsilon_{t}+\varepsilon_{x}\right) \end{array}$$

where $t_{0}$ and t are the initial and final tube wall thickness. Mises yield criterion and the associated isotropic hardening model are used in the current study; the effective strain $\varepsilon_{e}$ can be derived as

(3) $$\begin{array}{c} {\bar{\varepsilon }}_{e}=\frac{\sqrt{2}}{3}\sqrt{{\left(\varepsilon_{t}-\varepsilon_{\theta }\right)}^{2}+{\left(\varepsilon_{\theta }-\varepsilon_{x}\right)}^{2}+{\left(\varepsilon_{x}-\varepsilon_{t}\right)}^{2}} \end{array}$$

Based on the principle of energy balance, the external total power $J^{*}$ required in the hydro bulging process consists of the following terms, which can be expressed by the formula:

(4) $$\begin{array}{c} J^{*}=\dot{W_{i}}+\dot{W_{f}}+\dot{W_{b}} \end{array}$$

in which $\dot{W_{i}},\dot{W_{f}}$ and $\dot{W_{b}}$ represent internal deformation power, contact surface friction power and additional power, respectively. On a kinematically admissible velocity field with discontinuous lines S with sliding $\dot{U}$, they can be defined as [43]

(5) $$\begin{array}{cc} & J^{*}=2F_{t}\dot{U} \end{array}$$

(6) $$\begin{array}{cc} & \dot{W_{i}}=\frac{\bar{\sigma_{t}}\pi \left(d_{0}^{2}-d_{i}^{2}\right)}{2\sqrt{3}}\dot{U} \end{array}$$

(7) $$\begin{array}{cc} & \dot{W_{f}}=\frac{\bar{\sigma_{t}}\pi cd_{0}\left(L-D+h\right)}{\sqrt{3}}\dot{U} \end{array}$$

(8) $$\begin{array}{cc} & \dot{W_{b}}=\frac{P\pi d_{i}^{2}D}{2L}\dot{U} \end{array}$$

Substituting (5), (6), (7), (8)  into Eq. 4, an approximate formula to calculate the flow stress obtained by the hydraulic bulge test can be derived as

(9) $$\begin{array}{c} \bar{\sigma_{t}}=\frac{\sqrt{3}\left(4F_{t}L-P\pi d_{i}^{2}D\right)}{\pi L[d_{0}^{2}-d_{i}^{2}+2cd_{0}\left(L-D+h\right)]} \end{array}$$

in which $F_{t}$ is the total forming load and P is the internal fluid pressure. $d_{0}$ and $d_{i}$ are the initial outer and inner diameter of tested tubes. L is the original tube length and c is the shear friction coefficient. D is the axial punch displacement and h is the filling height.

It is evident that the flow stress and corresponding strain can be identified using the above equations based on the recorded experimental data such as axial feeding force, bulge height and so on. Hollomon isotropic hardening law is used to describe the strain-stress relationship of thin-walled aluminium alloy tubes, which can be written as

(10) $$\begin{array}{c} \bar{\sigma }=K{\bar{\varepsilon }}^{m} \end{array}$$

where K is the strength coefficient and m is the hardening exponent.

### 3.2 Inverse strategy

Inverse modelling techniques are widely used in the identification of material constitutive parameters for metal forming processes. They integrate FE simulations, optimization algorithms and actual physical experiments and can determine more accurate results by eliminating the mechanical and geometrical assumptions of classical analytical models. In this study, an automatic inverse framework has been developed to identify the material hardening parameters of aluminium alloy tubes.

**Fig. 7:**
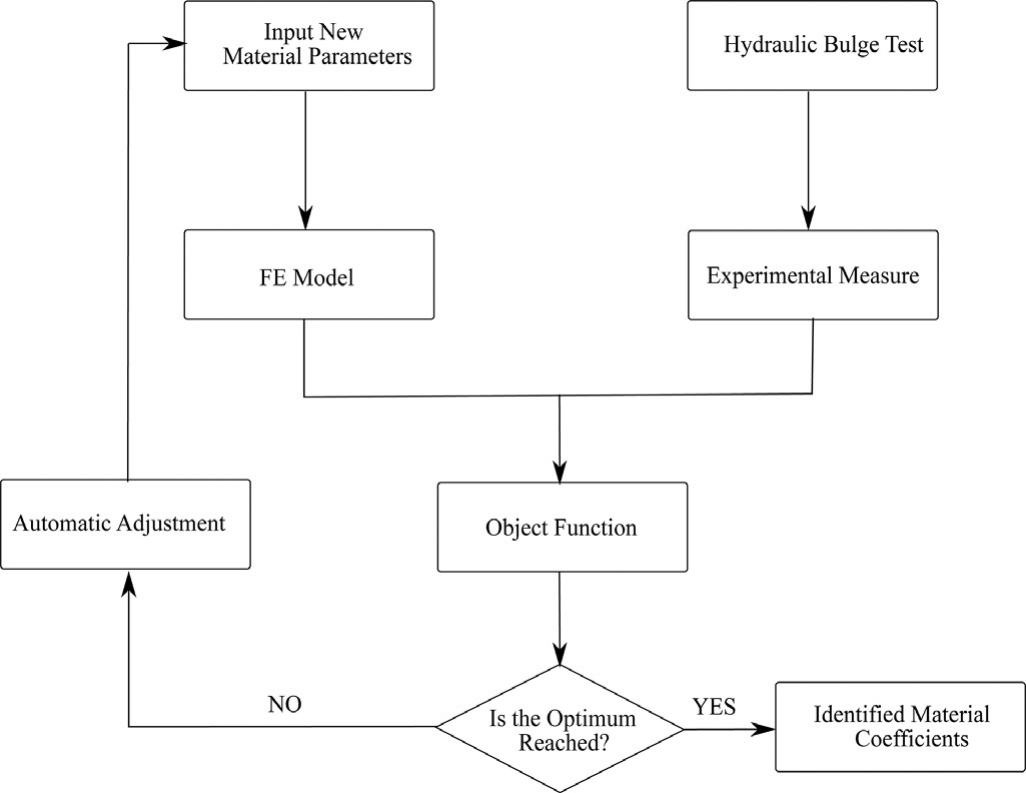
**Illustration of the flow chart of inverse strategy utilized in parameter identification based on hydraulic bulge test**.

The main principle behind the inverse analysis is to match experimental data from T-shape tube hydraulic bulge tests with FE simulation outputs. This fitting process is performed iteratively by adjusting design variables using the Levenberg-Marquardt algorithm. When the cost function based on the difference between experimental and simulated data is minimized, the iterative process will be terminated and the optimum solution is identified. Fig. 7 illustrates the flow chart of the inverse scheme utilized in parameter identification based on the T-shape tube hydraulic bulge test.

Objective functions are defined to evaluate the fitting quality between numerical results and experimental observations. Furthermore, a suitable cost function can determine more accurate material parameters and enable the optimization process to be more robust. The T-branch height, internal pressure, punch displacement and axial feeding force in the T-shape tube hydraulic bulge test are recorded as experimental data. The corresponding simulated results can be calculated by FE models. To correlate these two databases, the definition of the objective function follows the sum of least square errors, which can be written as

(11) $$\begin{array}{cc} & f_{1}=\beta_{1}f_{11}+\beta_{2}f_{12}+\beta_{3}f_{13} \end{array}$$

(12) $$\begin{array}{cc} & f_{11}=\sum_{p=1}^{n_{1}}{\left[\omega_{p}\left(h_{p}^{exp}-h_{p}^{sim}\right)\right]}^{2} \end{array}$$

(13) $$\begin{array}{cc} & f_{12}=\sum_{q=1}^{n_{2}}{\left[\omega_{q}\left(t_{q}^{exp}-t_{q}^{sim}\right)\right]}^{2} \end{array}$$

(14) $$\begin{array}{cc} & f_{13}=\sum_{r=1}^{n_{3}}{\left[\omega_{r}\left(F_{r}^{exp}-F_{r}^{sim}\right)\right]}^{2} \end{array}$$

in which $\beta_{1},\beta_{2},\beta_{3}$ are the scaling factors for different parts in the objective function, which satisfy $\beta_{1}+\beta_{2}+\beta_{3}=1$. F, h and t represent the axial feeding force, T-branch height and pole thickness, respectively. $n_{1},n_{2},n_{3}$ are the number of collected different types of experimental data. ω is the weighting coefficient for the pth point in the sub-objective function, which can be expressed as

(15) $$\begin{array}{c} \omega_{p}=M\frac{h_{p}^{exp}}{\sum_{p=1}^{n_{1}}\sum_{q=1}^{n_{2}}\sum_{r=1}^{n_{3}}\left(h_{p}^{exp}+t_{q}^{exp}+F_{r}^{exp}\right)} \end{array}$$

in which M is the total number of various experimental indicators, i.e., $M=n_{1}+n_{2}+n_{3}$. The other two weighting coefficients in the objective function can be expressed by similar formulas.

Hollomon’s power hardening model is used to describe the stress-strain behaviour of tubular materials in work hardening. The strength coefficient K and hardening exponent m in this equation are considered as the design variables, i.e., x=[K,m]. There are no special constraints on these two design parameters except that they must be greater than zero because the material behaviour should conform to the real physical world. This trust region scheme will be activated and ensure that all design variables are within a reasonable range once the new parameter to be solved exceeds the predetermined search space.

**Fig. 8:**
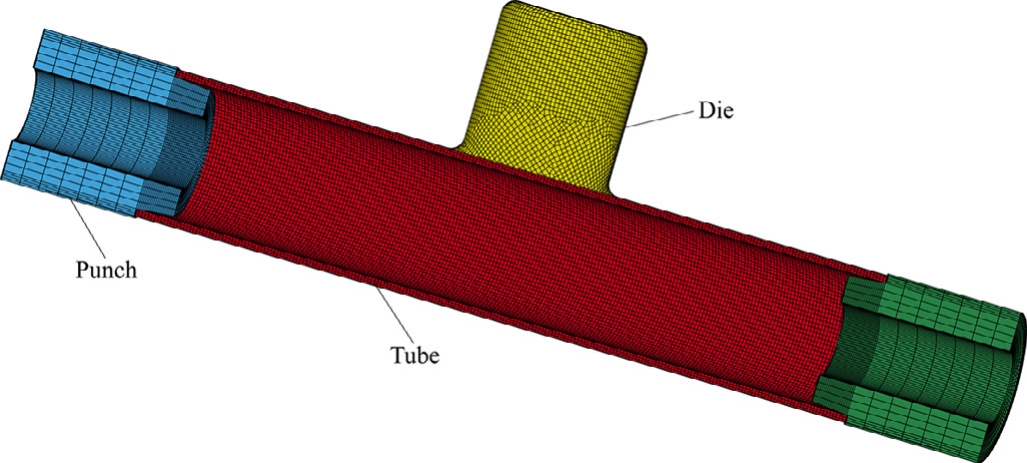
**FE model for the T-shape tube hydraulic bulge process**.

The FE model of the T-shape tube hydraulic bulge process is presented in Fig. 8. The tube, punches and dies constitute the entire model where 3D brick elements with eight nodes are assigned to the first two parts, and the latter part is set as a rigid body with four nodes 2D shell elements. The type and size of tubular materials used in the model are consistent with the actual test and these details are shown in Section 2.1. Mises yield criterion and isotropic power hardening law denote the material stress-strain behaviour. In the simulation, the movement of two punches and internal fluid pressure curves follow the loading path recorded in the actual experiment, as shown in Fig. 4. The friction between the workpiece and dies is described by Coulomb law with 0.1 of the friction coefficient. To reduce the computation time, the mass scaling factor is introduced into the FE model. Moreover, due to the symmetry of the geometry, material properties and loads, a half of FE model for the whole assembly is constructed to improve calculation efficiency as well.

The inverse problem can be considered as an optimization problem to be solved, and an improved Levenberg-Marquardt algorithm [44] is constructed to minimize the objective function defined in Eq. 11 with respect to the design variable x=[K,m] subject to specific constraints. The ellipsoidal trust region scheme is introduced into the Levenberg-Marquardt algorithm to solve the approximated model and can remove the influence of poor scaling problems in the numerical optimization, where the magnitude of design variables, i.e., the strength coefficient and hardening exponent, has different orders.

Identified material parameters from the tensile test are chosen as the starting point for the inverse model, then a new point can be defined as

(16) $$\begin{array}{c} x_{new}=x+\left[J{\left(x\right)}^{T}J\left(x\right)+\mu D{\left(x\right)}^{T}D\left(x\right)\right]J{\left(x\right)}^{T}f\left(x\right) \end{array}$$

where J(x) is the Jocabian matrix of the objective function at the current point and can be obtained using the finite difference method. D(x) is a diagonal matrix that enables the algorithm invariant and is calculated based on the information from the first derivative of the objective function. The damping parameter μ can control the searching direction and step size for the next iteration.

During the optimization, material parameters are updated by the Eq. 16 step by step. When the gradient of the objective function or the change of x is less than a small positive constant ϵ given by users, the iteration process will be terminated. Moreover, a maximum iteration number $k_{max}$ is defined as a safeguard to avoid an infinite loop and the stopping criteria can be expressed as

(17) $$\begin{array}{cc} & ∥x_{new}-x∥\leq \epsilon_{1}(∥x∥+\epsilon_{1}) \end{array}$$

(18) $$\begin{array}{cc} & ∥J{\left(x_{new}\right)}^{T}f\left(x_{new}\right)∥\leq \epsilon_{2} \end{array}$$

## 4 Results and discussion

**Fig. 9:**
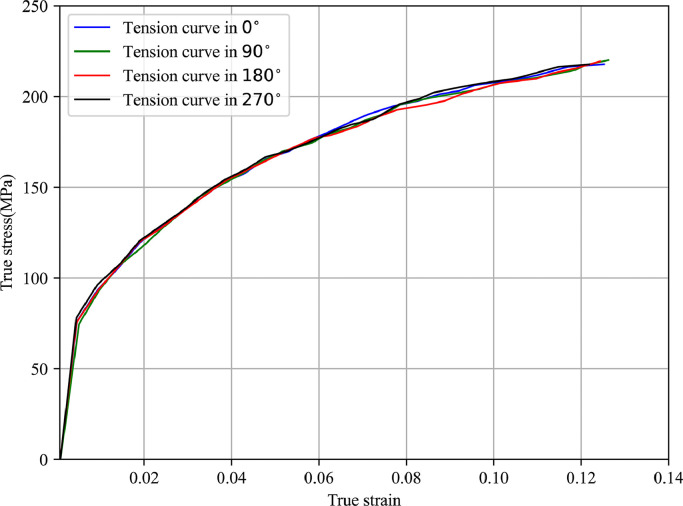
**True stress-strain curve determined by tensile test for 5049-O aluminium alloy**.

**Fig. 10:**
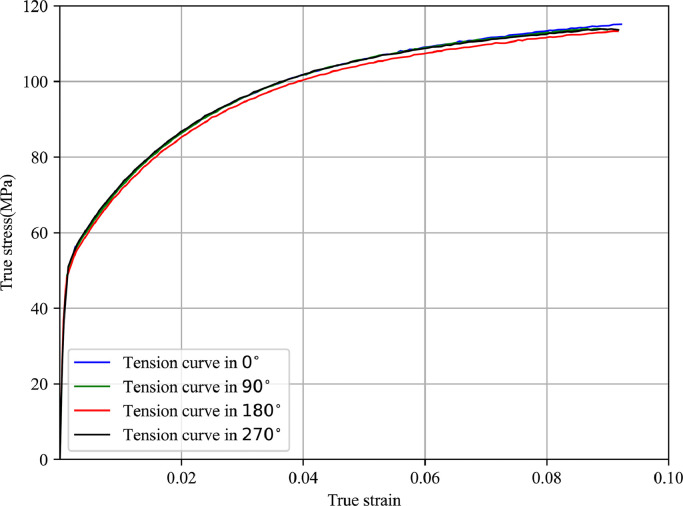
**True stress-strain curve determined by tensile test for 6060-O aluminium alloy**.

In this section, the obtained experimental results and their comparisons will be discussed. The recorded tensile data is translated into the true strain-stress curves and corresponding material parameters can be determined using the least square fitting method. Figs. 9,  10 present the true stress-strain curves identified by tensile tests for 5049-O and 6060-O aluminium alloys. The difference among stress-strain curves at different circumferential positions is so small that it can be ignored. It can be concluded that the two types of materials show strong isotropic features after the fully annealing heat treatment process, and the isotropic power hardening law can be used to describe the deformation behaviour in the forming process. Tables 1,  2 present the material property such as yield stress, ultimate strength and fitted constitutive coefficients for aluminium alloys 5049-O and 6060-O, which show consistent results with the above Figs. 9,  10 as well.

**Table 1:** **Material constitutive parameters identified by tensile test for 5049-O aluminium alloy**.

Circumferential position	Yield strength(MPa)	Ultimate tensile strength(MPa)	Elongation(%)	Strength coefficient (MPa)	Hardening exponent
0${}^{\circ }$	73.36	226.47	12.94	431.52	0.323
90${}^{\circ }$	73.69	227.70	13.04	433.21	0.324
180${}^{\circ }$	69.22	224.61	13.37	431.38	0.325
270${}^{\circ }$	70.50	223.20	13.10	432.36	0.321
Mean value	71.69	225.50	13.11	432.12	0.323

**Table 2:** **Material constitutive parameters identified by tensile test for 6060-O aluminium alloy**.

Circumferential position	Yield strength(MPa)	Ultimate tensile strength(MPa)	Elongation(%)	Strength coefficient (MPa)	Hardening exponent
0${}^{\circ }$	54.88	115.40	9.68	201.94	0.221
90${}^{\circ }$	54.58	114.01	8.95	202.99	0.223
180${}^{\circ }$	53.73	114.00	9.78	199.48	0.222
270${}^{\circ }$	54.72	113.90	9.16	202.65	0.222
Mean value	54.48	114.33	9.39	201.77	0.222

**Fig. 11:**
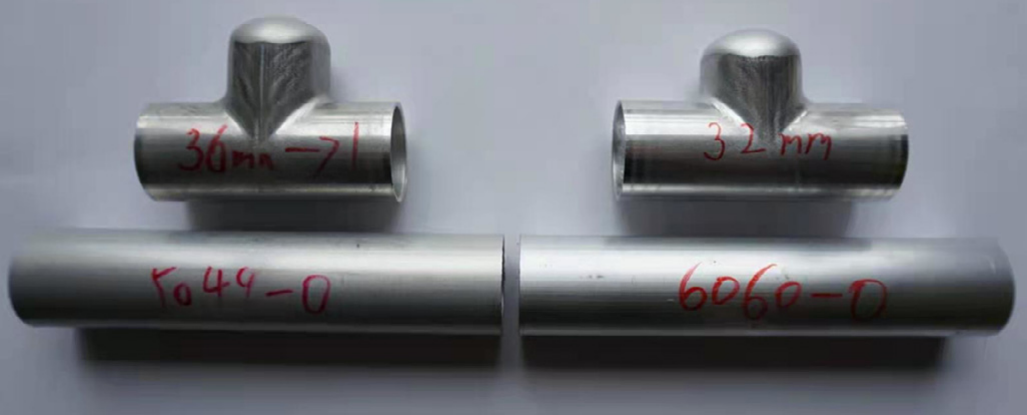
**Hydro bulged specimens before and after test for aluminium alloy 5049-O and 6060-O**.

The original and bulged tubular components for aluminium alloys 5049-O and 6060-O are presented in Fig. 11. It can be observed that these deformed samples have no fracture and wrinkling, which means the defined loading path can produce perfect components and generate the reasonable experimental databases for the parameter identification process. Tables 3, 4 show the part of the measured data at different bulging stages for aluminium alloys 5049-O and 6060-O. The developed inverse strategy is used to determine the strength coefficient and hardening exponent by reducing the difference between the experimental data and simulated predictions.

**Table 3:** **Typical experimental data obtained by T-shape tube hydraulic bulge test for 5049-O aluminium alloy**.

Stage No.	Punch displacement(mm)	Internal pressure(MPa)	Filling height(mm)	Axial feeding force(kN)
1	1.04	0.39	0.17	28.33
2	5.15	15.94	4.70	50.23
3	9.27	18.60	8.17	59.04
4	13.40	20.65	10.79	68.03
5	17.51	22.52	13.44	76.83
6	21.64	24.20	16.28	84.92
7	25.75	26.63	19.36	93.99
8	29.85	29.34	22.66	103.28
9	36.02	34.23	28.94	119.21

**Table 4:** **Typical experimental data obtained by T-shape tube hydraulic bulge test for 6060-O aluminium alloy**.

Stage No.	Punch displacement(mm)	Internal pressure(MPa)	Filling height(mm)	Axial feeding force(kN)
1	1.06	6.70	0.32	32.39
2	4.13	10.91	3.90	43.42
3	7.24	11.71	6.39	49.52
4	10.34	12.63	8.01	54.72
5	13.43	13.56	9.81	58.68
6	19.64	15.98	13.52	68.76
7	25.86	18.48	18.17	76.23
8	32.01	20.07	23.39	84.53

**Fig. 12:**
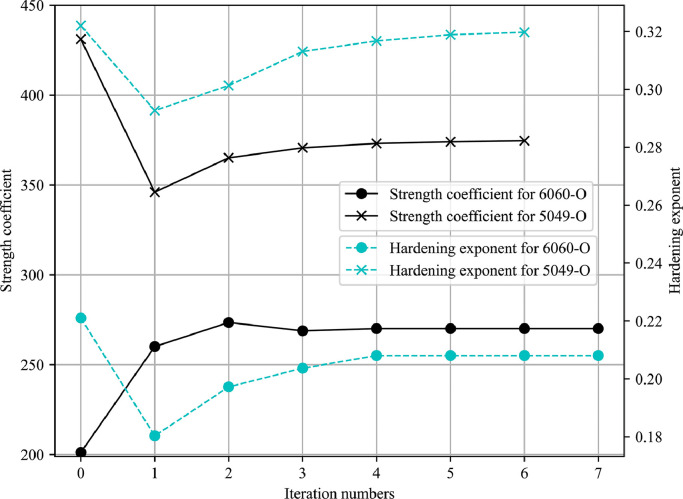
**Iteration history of two design variables**.

**Fig. 13:**
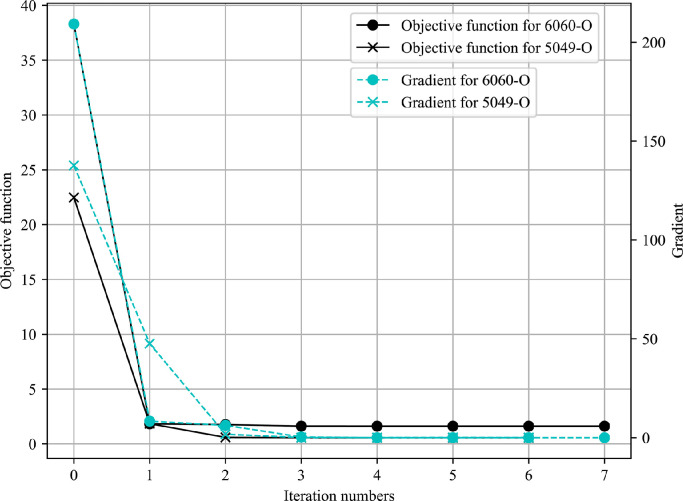
**Iteration history of the objective function and its gradient**.

The iteration history of two design variables is shown in Fig. 12, where the identified parameters from the tensile test are chosen as the initial points. Fig. 13 illustrates the changing process of the objective function and its gradient during the automatic identification. As the results indicate, the strength coefficient K and hardening exponent m gradually converge to the optimal value while the objective function and its gradient are reduced to the lower level close to zero after fewer iterations, which proves that the inverse modelling strategy can be applied to identify material parameters for the T-shape tube hydraulic bulge process with axial feeding force and it shows a good performance in terms of the robustness and efficiency.

The other analytical model described in Section 3.1 is also selected as a post-processing procedure to fit the experimental data using the power hardening law. The material parameters identified by the theoretical model and other methods are presented in Table 5. It can be observed that the values of strength coefficient and hardening exponent of 5049-O and 6060-O aluminium tubes determined by the bulge test and tensile test have a large discrepancy because the strain range in the uniaxial tensile test is 10%−20%, but it can be higher than 100% in the hydraulic bulge test. Moreover, material parameters calculated by the inverse strategy and analytical model based on the bulge test are quite different, and one reason for this is that the inverse scheme combines the incremental theory with the gradient-based optimization algorithm while the theoretical model is based on membrane theory with geometrical and mechanical assumptions.

**Table 5:** **Comparison of identified constitutive parameters based on bulge test and tensile test**.

Experimental type	6060-O aluminium alloy		5049-O aluminium alloy	
	Strength coefficient (MPa)	Hardening exponent	Strength coefficient (MPa)	Hardening exponent
Bulge test				
Inverse scheme	270.63	0.201	374.60	0.320
Analytical method	212.47	0.350	403.06	0.460
Tensile test	201.77	0.222	432.12	0.323

**Fig. 14:**
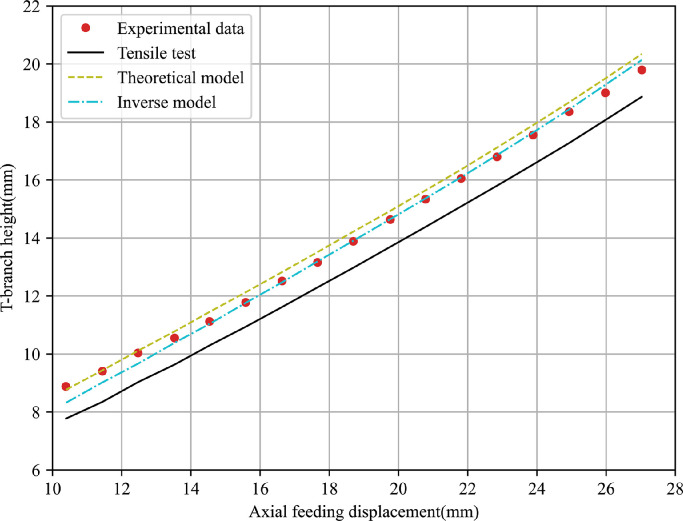
**Comparison of FE outputs and experimental data of punch displacement versus T-branch height for 5049-O aluminium**.

**Fig. 15:**
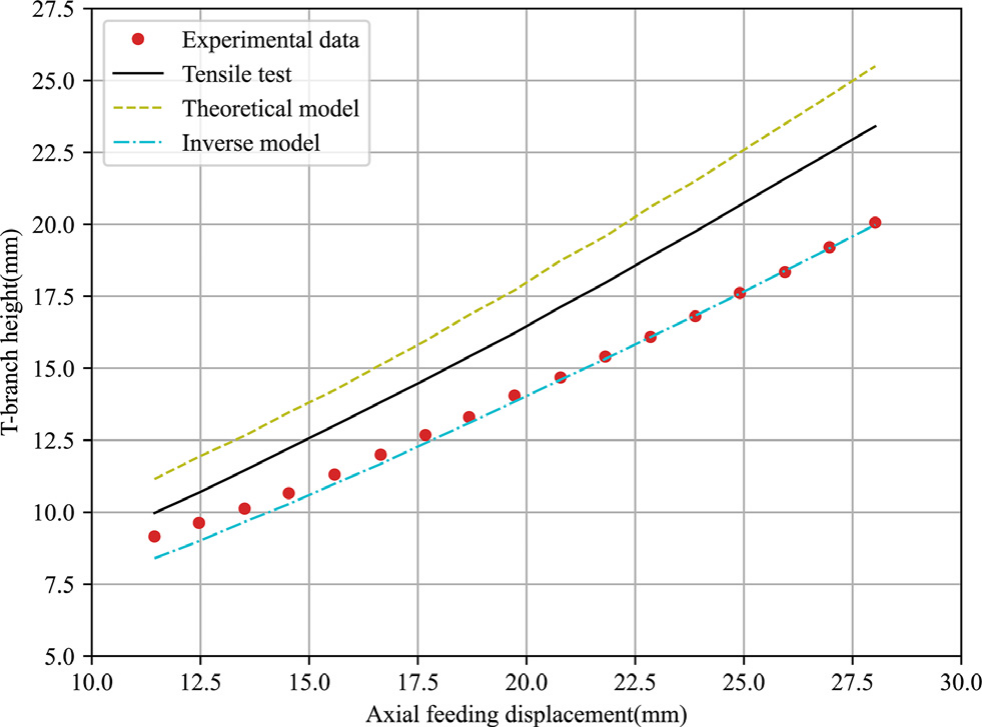
**Comparison of FE outputs and experimental data of punch displacement versus T-branch height for 6060-O aluminium**.

To evaluate the accuracy of the obtained results, T-shape tube hydroforming processes under different loading paths are performed for 5049-O and 6060-O thin-walled aluminium alloys, and corresponding FE simulations are conducted using material parameters obtained by above three methods. The predicted T-branch height and axial feeding force are used to compare with that recorded during the experiments. Figs. 14,  15 present the comparison of punch displacement versus T-branch height between FE simulated outputs and experimental measured values for 5060-O and 6060-O aluminium.

**Fig. 16:**
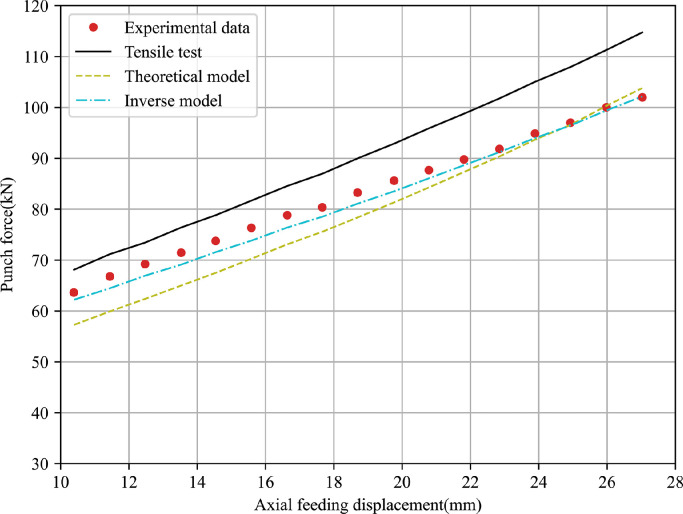
**Comparison of FE outputs and experimental data of punch displacement versus axial feeding force for 5049-O aluminium**.

**Fig. 17:**
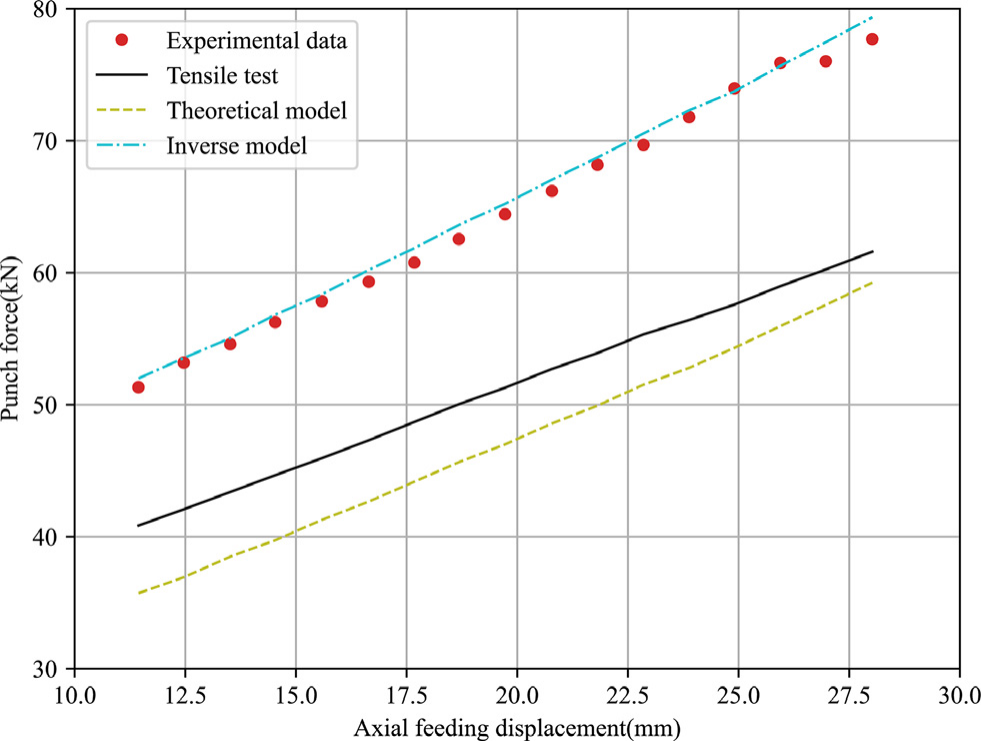
**Comparison of FE outputs and experimental data of punch displacement versus axial feeding force for 6060-O aluminium**.

It can be observed that the predicted bulge height using material parameters obtained from the tensile test differs greatly with the experimental values. Further, FE outputs with the inverse model based on the bulge test have the best agreement with the experiment data among them while the analytical model leads to a slight increase of the fitting error. The comparison of punch displacement versus axial feeding force between FE predictions and experimental measurements for two materials is shown in Figs. 16,  17. The inverse strategy based on bulge tests shows a better fitting quality compared with the analytical model and tensile test. A similar phenomenon is due to the maximum effective strain before necking in the tensile test being lower than that in the bulge test and the biaxial stress state in the bulge test being closer to the one in the actual hydroforming process.

**Fig. 18:**
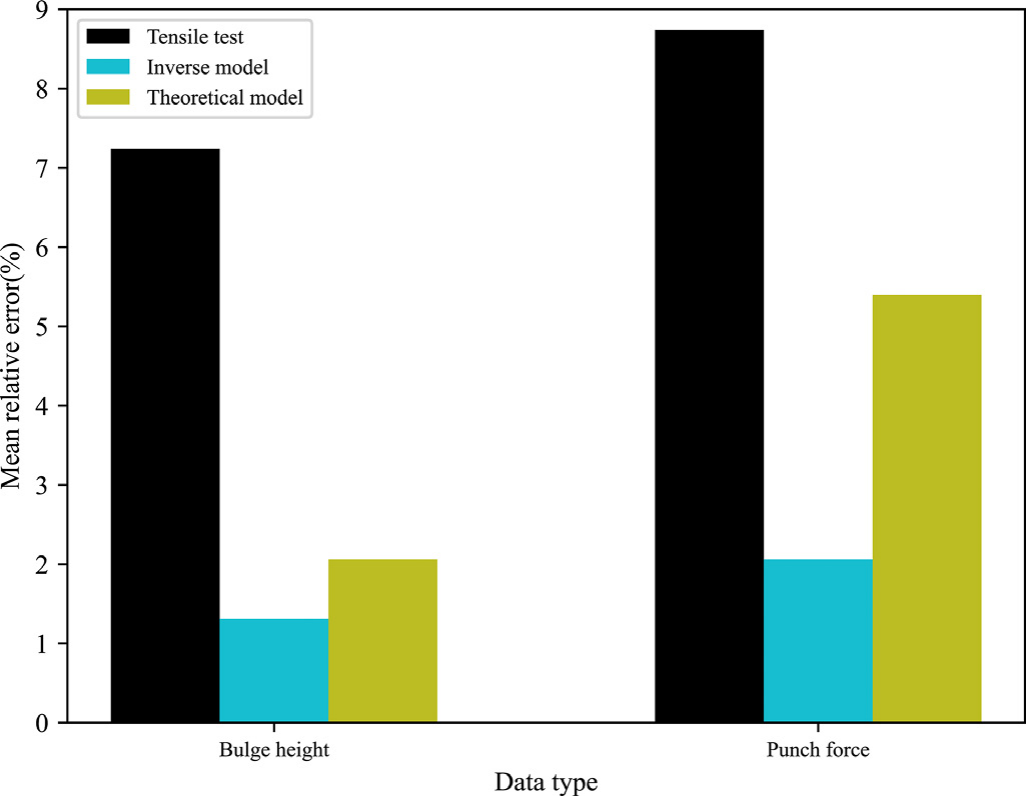
**Different mean errors obtained by three methods for 5049-O aluminium**.

**Fig. 19:**
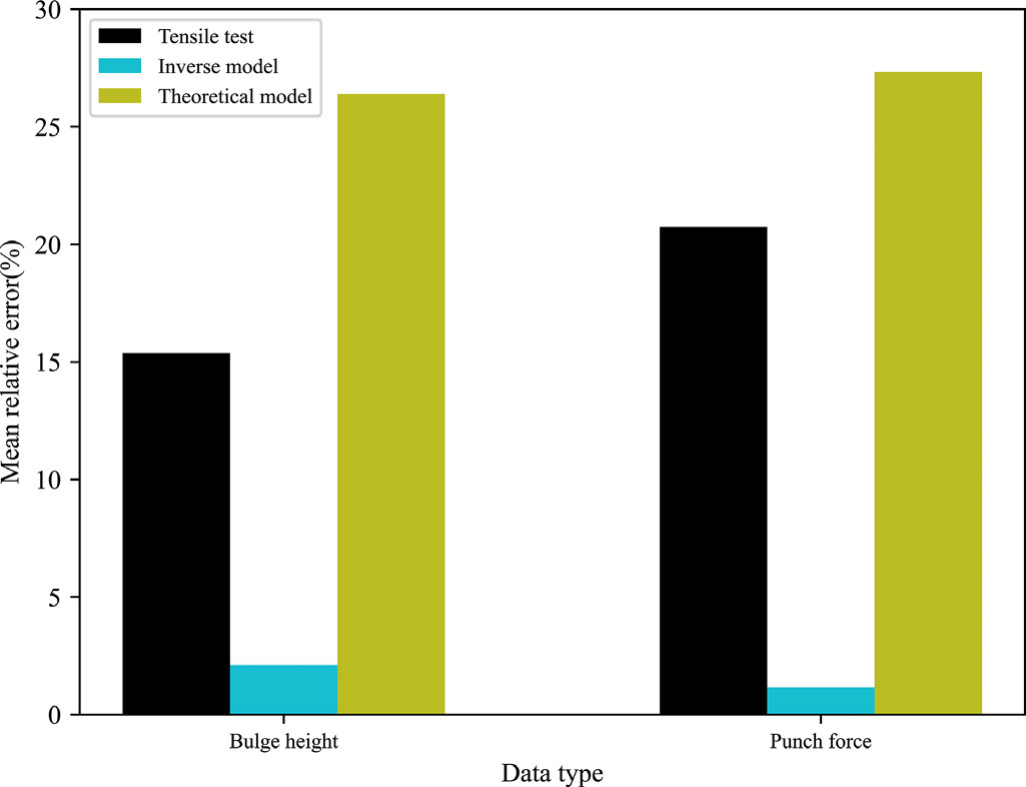
**Different mean errors obtained by three methods for 6060-O aluminium**.

For more in-depth comparisons, the resulting error between FE predictions and experimental measurements is quantified using the maximum, mean and minimum of variation values, where the average value of the relative deviation can be expressed as $\Psi =\frac{1}{N}\sum_{i=1}^{N}\left(\frac{\left(D_{exp}^{i}-D_{sim}^{i}\right)}{D_{exp}^{i}}\right)$. N is the total number of experimental points. $D_{exp}^{i}$ and $D_{sim}^{i}$ represent the experimental and simulated results, respectively. Table 6 summarises the quantitative comparisons of all types of experimental data for 5049-O and 6060-O aluminium alloys. Figs. 18,  19 graphically show the most representative mean relative error obtained by different methods.

**Table 6:** **Quantitative comparisons between FE predictions and experimental values for 5049-O and 6060-O aluminium alloys**.

Data type	Reference	Model	5049-O aluminium			6060-O aluminium		
			Max (%)	Mean (%)	Min (%)	Max (%)	Mean (%)	Min (%)
Bulge height	Experiment	Tensile test	12.46	7.24	4.68	17.68	15.37	8.98
		Inverse model	6.31	1.31	0.12	8.25	2.11	0.003
		Theoretical model	2.99	2.06	0.28	28.13	26.40	21.85
Punch force	Experiment	Tensile test	12.52	8.74	6.11	22.29	20.74	19.88
		Inverse model	3.49	2.06	0.19	2.13	1.15	0.30
		Theoretical model	10.30	5.40	0.32	30.57	27.33	23.74

From the results presented, it is evident that the mean deviations of 7.24% of the T-branch height and 8.74% of the punch force predicted by the tensile test are higher than corresponding values of 2.06% and 5.40% obtained by the analytical model for the 5049-O aluminium alloy. For the 6060-O aluminium alloy, the theoretical model presents a larger relative error to the experimental data compared with the tensile test with respect to the bulge height and compressive force. It is worth pointing out that the inverse model produces the smallest fitting gap to the measured bulge height and punch force for two types of tubular materials. One possible reason is that the hydraulic bulge test reproduces similar loading conditions to the tube forming process, and the inverse model based on the incremental theory eliminates excessive assumptions in the classical theoretical model.

## 5 Conclusion

To evaluate stress-strain characteristics of tubular materials at the large strain range, hydraulic bulging tests under axial compressive force have been performed. An intelligent inverse strategy integrating FE simulations with gradient-based optimization algorithms is proposed to process and analyze the collected T-branch height, punch displacement and axial feeding force during the experiment. The determined material parameters using the inverse scheme based on the novel bulge test are compared with that obtained by the tensile test and a theoretical analysis. The main conclusions can be drawn as follows:

(1) The values of the strength coefficient and hardening exponent of EN AW 5049-O and 6060-O aluminium tubes calculated by the above three methods are different, and the hydraulic bulge test can characterize the mechanical properties of tubular materials in the range of larger strains and reduce the extrapolation of stress-strain data in FE simulations compared with the uniaxial tensile test.

(2) The proposed automatic inverse identification framework can be extended to the tube hydraulic bulge test under axial compressive force, and its capabilities for post-processing experimental resources have been verified by characterizing two different types of thin-walled tubes. The analytical model is a simple method to determine the stress-strain data from the bulge test compared with the complex inverse strategy and can sometimes improve the results accuracy compared with that from the uniaxial tensile test.

(3) Predicted bulge height and axial feeding force from FE simulations using material parameters identified by the inverse modelling technique, analytical model based on the bulge test and uniaxial tensile test are used for comparison with the experimental data. The results show that the inverse model leads to the smallest fitting error between numerical and experimental values, which means the intelligent inverse identification framework is the most accurate post-processing procedure to characterize mechanical properties of tubular materials.

## Data availability statement

The raw data required to reproduce these findings can be available from the corresponding author upon reasonable request.

## Declaration of competing interest

The authors declare that they have no conflicts of interest in this work.
